# Anticonvulsants and antipsychotics for treating anxiety and depressive symptoms in people with alcohol dependence: a systematic review

**DOI:** 10.1192/bjo.2021.801

**Published:** 2021-06-18

**Authors:** Ka Yee Chow, Katy Jones, Musa Sami

**Affiliations:** University of Nottingham

## Abstract

**Aims:**

Anxiety and depressive symptoms are frequent in people with alcohol use disorders (AUDs) (approximately 55%) and are associated with worse outcomes. Current interventions to treat mood symptoms in alcohol-dependent adults, including psychosocial therapies, anxiolytics and antidepressants have shown limited benefits to date. The efficacy of anticonvulsants and antipsychotics have been studied but never previously systematically reviewed. We aimed to assess the efficacy of anticonvulsants and antipsychotics in treating anxiety and depressive symptoms in alcohol-dependent adults.

**Method:**

A literature search of MEDLINE, EMBASE and PsycINFO was performed through October 2020 to identify all English-language articles of double-blinded randomised controlled trials that included adults with AUDs who were treated with an anticonvulsant or antipsychotic for at least four weeks. A combination of search terms was used to describe the AUDs, study medications and the primary outcome. Participants with other psychiatric disorders were excluded. Mean changes in anxiety and depressive scores were evaluated in addition to the adverse events and withdrawal rates. The risk of bias of each study was also assessed.

**Result:**

Of 393 citations identified, 23 studies (2823 participants) met the inclusion criteria. Eighteen studies examined ten different anticonvulsants, while five studies examined two antipsychotics. The heterogeneity between the trials' methodology led to conflicting results; however, as the low-quality trials were excluded, the majority agreed that anticonvulsants and antipsychotics were not superior in moderating anxiety and depressive symptoms of alcohol-dependent adults. All antipsychotics were safe and well-tolerated, but adverse events were associated with several anticonvulsants (high dose baclofen, gabapentin, topiramate and valproate).

**Conclusion:**

Current information on anticonvulsants and antipsychotics is insufficient to extrapolate the benefits of treating anxiety and depressive symptoms in alcohol-dependent adults. Further research must be conducted into improving the quality of reporting and understanding the comorbidity's underlying mechanism, and alternative treatment approaches.

